# Contact tracing management of a measles case in a paediatric hospital: experience of the local public health unit, Portugal, January 2024

**DOI:** 10.2807/1560-7917.ES.2024.29.17.240022

**Published:** 2024-04-25

**Authors:** Daniel Saldanha Resendes, António Tomás, Mariana Pinção Cardoso, Sebastian von Schreeb, Rita Miranda Ferrão, Paula Vasconcelos, Maria Helena Almeida, Bruno Novo Castro, Vasco Ricoca Peixoto, Renato Lourenço Silva, Margarida de Morais

**Affiliations:** 1 Public Health Unit, Local Health Unit of São José, Ministry of Health, Lisbon, Portugal; 2 Center for Public Health Emergencies, Directorate-General of Health, Lisbon, Portugal; 3 Directorate of Information and Analysis, Directorate-General of Health, Lisbon, Portugal; 4 ECDC Fellowship Programme, Field Epidemiology path (EPIET), European Centre for Disease Prevention and Control (ECDC), Stockholm, Sweden

**Keywords:** Measles, Contact Tracing, MMR vaccine, Lisbon, Portugal, Paediatric, Hospital, Epidemiological Surveillance, Communicable Diseases, Public Health Unit

## Abstract

In January 2024, a child was diagnosed with measles in a paediatric hospital in Lisbon. Of 123 contacts, 39 (32%) were not fully immunised, presenting a risk for a potential outbreak. The public health unit initiated control measures and identified challenges during the response, such as the lack of interoperability between information systems and accessing vaccination records. The lessons learned prompted changes to national contact tracing procedures for measles, further strengthening Portugal’s preparedness.

Measles has been considered eliminated in Portugal since 2015 [1], and the country has maintained a vaccination coverage ≥ 95% for two doses of the measles, mumps, rubella (MMR) vaccine for the last two decades [2]. Recently, there have been two major outbreaks, one in 2017 and another in 2018; both originated from imported cases [2].

In this article we describe the contact tracing methodology adopted after a confirmed case of measles was detected and present a descriptive analysis and evaluation of the implemented control measures.

## Event description

In January 2024, the health authority of our Public Health Unit (PHU) was informed of a suspected case of measles. The alert was given by the on-duty emergency room (ER) physician at a major paediatric hospital in Lisbon, Portugal. The case was an unvaccinated child who had travelled with their family to Lisbon from a city in the United Kingdom (UK) where an outbreak of measles was ongoing at that time [3]. The child’s symptoms began 1 day after arrival to Lisbon and included fever, malaise and, later, a rash that prompted medical observation in the ER 3 days later. The health authority from our PHU provided guidance on the procedures regarding isolation and collection of biological samples (blood, urine and saliva), which were sent to the national reference laboratory (Instituto Nacional de Saúde Dr Ricardo Jorge (INSA)). The case was laboratory-confirmed 4 days after symptom onset.

Immediately after the alert, the epidemiological inquiry was initiated by a PHU from another municipality in the Lisbon area where the family was staying. The inquiry gathered information about the family members, details of the family's flight from the UK to Portugal and a list of places visited by the case during the infectious period (a civil registry office, two shopping malls and the ER of the hospital). According to the national programme for measles elimination [4], the alert triggered the chain of command at regional and national level to respond to the imported case.

## Contact tracing

### Definition of contact

A contact was defined as anyone who shared the same living space with the case for any length of time or stayed in the same setting until 2 h after the case had left, between the date of arrival in Portugal and 5 days later when the case was isolated [4,5].

### Contact management responsibility

Since the locations visited by the case fell under the jurisdiction of different PHU, contact tracing for each site was assigned to its responsible PHU. Our PHU was responsible for contact tracing in the civil registry office and in the ER of the paediatric hospital (paediatric patients and their caregivers/companions).

### Contact prioritisation and control measures implemented by the Public Health Unit

Two days after the case’s admission to the hospital, our PHU received a list of all paediatric patients present in the ER waiting room from the time of arrival of the case until 2 h after they left. This list contained name, unique health user number, phone number and date of birth for each patient. Using this information, we assessed the contacts’ immunisation status using the national electronic vaccination registry and contacted them by phone and email. Considering that we received the list of patients 2 days after the exposure, on a Friday, and that we only had access to age and immunisation status before calling, we used the following prioritisation criteria to select those we would call first based on who was most vulnerable to severe disease: (i) infants younger than 6 months, (ii) infants 6–12 months-old, (iii) children 1–5 years-old and (iv) anyone without documented previous full immunisation against measles [6,7]. Contacts were considered as fully immunised whenever they presented a documented history of previous measles infection or two doses of any measles vaccine. Patients and their guardians were informed of their exposure to measles and when not fully immunised, they were instructed on how to receive post-exposure prophylaxis with the MMR vaccine or intravenous human non-specific immunoglobulin (IgIV) within 72 h or 6 days, respectively, from first contact with the confirmed case. In addition, all contacts were advised to call the National Health Service hotline in case they developed symptoms suggestive of measles. The PHU São José contacted vaccination units to ensure MMR vaccines were available and that contacts would get vaccinated within the 72 h period. The contacts needing IgIV were referred to the paediatric hospital’s infectious diseases service for its administration.

### Data analysis

For the descriptive analysis, we included details from all hospital contacts, incorporating details from patients, guardians and healthcare professionals. We identified 123 contacts, 82 were female (67%) and 41 male (33%) (Table 1). Regarding age distribution, 74 were adults (60%) and 49 were children (40%), of whom eight were younger than 12 months. Regarding previous immunisation status, 39 (32%) individuals were not fully immunised, only one of them was a healthcare professional. None of the identified contacts became a confirmed or suspected case of measles.

**Table 1:** Characteristics of the contacts of a measles case, Portugal, January 2024 (n = 123)

Characteristics	Completed vaccination n = 70		No evidence of prior immunisation n = 39		Past infection n = 14		Total n = 123	
	n	%	n	%	n	%	n	%
Sex								
Female	48	69	23	59	11	79	82	67
Male	22	31	16	41	3	21	41	33
Age group								
< 6 months	0	0	5	13	0	0	5	4.1
6–12 months	0	0	3	7.7	0	0	3	2.4
1–5 years	1	1.4	14	36	0	0	15	12
6–18 years	24	34	2	5.1	0	0	26	21
> 18 years	45	64	15	38	14	100	74	60
Contact type								
Hospital contact (guardian)	16	23	18	46	6	43	40	33
Hospital contact (patient)	28	40	20	51	0	0	48	39
Hospital contact (HCW)	26	37	1	2.6	8	57	35	28

HCW: healthcare workers.

Table 2 presents the results of the measures implemented to guarantee prompt post-exposure immunisation. Among the 39 contacts identified as susceptible to infection, 23 contacts received immunisation within the recommended time (72 h from the exposure in case of vaccination, and 6 days from the exposure in case of IgIV administration). All eligible contacts younger than 12 months were immunised on time.

**Table 2:** Results of immunisation efforts among contacts of an imported measles case, by age group, Portugal, 2024 (n = 39)

Age group	Eligible for immunisation	IVIG < 6 days	Vaccinated < 72h	Vaccinated > 72h	Immunised on time	
		n	n	n	n	%
< 6 months	5	5	0	0	5	100
6–12 months	3	0	3	0	3	100
1–5 years	14	0	9	3	9	64.3
6–18 years	2	1	0	0	1	50.0
> 18 years	15	0	5	5	5	33.3
**Total**	**39**	**6**	**17**	**8**	**23**	**59.0**

IVIG: intravenous human non-specific immunoglobulin.

## Discussion

We found that 17% of the contacts 6 years or older were not immunised, which illustrates that also in countries with high vaccination coverage such as Portugal [2], there are pockets of the population with vaccine coverage below the herd immunity threshold, which incurs the risk of an outbreak upon introduction of infected cases. To address this risk, quick identification and immunisation of contacts of imported cases is paramount.

Of 39 susceptible contacts, more than half received immunisation on time. Challenges in obtaining prompt identification of contacts from the hospital and access to vaccination sites over the weekend were among the factors hindering timely immunisation. Furthermore, our efforts were hampered by the lack of integration between information systems, particularly when accessing the vaccination status of individuals residing outside our jurisdiction. This issue accentuated that improved system interoperability is needed to enhance the efficiency of our public health response. Finally, we recognised the need for better coordination between the PHU and the vaccination points to ensure that the patients were properly and quickly admitted for post-exposure prophylaxis.

We adopted a sensitive contact definition for those exposed since this was a high-risk setting with potentially many not fully immunised children, and also because Portugal had until then only had one case of measles since 2021 [8]. We implemented a prioritisation system that focused on those most vulnerable to severe disease considering that we started calling patients on a Friday and had limited availability of vaccination sites over the weekend. All children younger than 12 months were immunised on time with vaccine or immunoglobulin. We believe that other Public Health Units should adopt such a system when faced with a large number of contacts and a limited time window to ensure that those at highest risk are prioritised. Three months after the confirmation of the index case, there have been no confirmed or suspected measles cases among our identified contacts, which indicate that our response may have mitigated broader transmission.

The local team's insights prompted the Directorate General of Health in Portugal to update measles contact tracing methods in the national epidemiological surveillance system (SINAVE). A system was implemented that links individuals' health identification numbers and the national vaccination registry, enabling the automatic import of contacts' immunisation status to SINAVE.

With recent measles outbreaks being reported throughout Europe [9,10], it has been important to reinforce Portugal’s capacity to maintain the measles post-elimination status. We expect that our current experience with measles contact tracing management can contribute to better preparedness and response efforts for future measles alerts at the national and international level.

## Conclusion

Considering the resurgence of new cases of measles throughout Europe, more public health teams might be faced with similar circumstances such as this one. Our work has shown that even with limited vaccination sites and time for effective post-exposure prophylaxis, it is possible to protect the ones most vulnerable to severe disease by following a prioritisation system.
